# Fabrication and Performance Evaluation of Multi-Stimuli-Responsive Hydrogels Constructed from Hyperbranched Skeletons

**DOI:** 10.3390/gels12080694

**Published:** 2026-08-04

**Authors:** Xue Wang, Jun Wang, Gen Li, Yang Zhao, Zhihua Guo, Keliang Wang

**Affiliations:** 1 Key Laboratory for EOR Technology (Ministry of Education), College of Petroleum Engineering, Northeast Petroleum University, Daqing 163318, China; 2 Provincial Key Laboratory of Polyolefin New Materials, College of Chemistry and Chemical Engineering, Northeast Petroleum University, Daqing 163318, China; 3 School of Earth Sciences, Northeast Petroleum University, Daqing 163318, China

**Keywords:** broom-shaped hyperbranched polymers, hydrogels, low-permeability reservoirs, swelling behavior, responsiveness

## Abstract

Long-lasting, high-strength plugging materials are required for deep profile control in low-permeability fractured reservoirs. In this study, a series of hyperbranched copolymer hydrogels was prepared through an aza-Michael addition-amidation-one-pot end-group coupling strategy. Linear alkylamines were used as cores to synthesize amino-terminated generation 1.0 G broom-shaped hyperbranched macromolecular backbones via a divergent route. The resulting backbones were subsequently crosslinked with linear α,ω-diepoxy-terminated poly(ethylene glycol), affording three structurally well-defined hydrogels, denoted as C2HG, C6HG, and C8HG. Structural and physicochemical characterization showed that all hydrogels possessed interconnected three-dimensional porous networks, good thermal stability, and a lower critical solution temperature of approximately 37 °C. Rheological analysis demonstrated predominantly elastic behavior, with the storage modulus (G′) consistently exceeding the loss modulus (G″), together with pronounced shear-thinning characteristics favorable for injection into deep, low-permeability formations. By varying the alkyl-chain length of the hyperbranched backbone, the balance between environmental tolerance and plugging performance could be effectively regulated. These findings establish a structure–property relationship between backbone hydrophobicity and hydrogel performance and demonstrate that PEG-crosslinked hyperbranched copolymer hydrogels are promising candidates for deep-profile control and water shutoff in high-salinity, low-permeability fractured reservoirs.

## 1. Introduction

With the continuous development of oil and gas resources, low-permeability fractured reservoirs have become an important strategic area for increasing oil reserves and production in China. However, such reservoirs generally have problems of low porosity, low permeability, and uneven fracture development [1,2]. During the long-term water injection development process, injected water can easily flow along highly permeable fractures or large pores, resulting in a significant reduction in water flooding sweep efficiency and serious damage to economic benefits [3]. Therefore, developing an efficient water plugging and profile control system that can effectively plug large fractures and achieve deep profile control and fluid diversion is a key technical problem to ensure the efficient development of low-permeability oilfields.

At present, conventional polymer gels and pre-crosslinked particulate gels (PPG) have been widely used for fracture sealing [4,5,6]. However, field application practices have shown that many traditional gel systems have obvious inherent limitations. On the one hand, polyacrylamide crosslinked gels are prone to irreversible mechanical degradation during pumping at high temperatures and high shear rates [7,8]. On the other hand, in highly mineralized formation water (especially when it contains a large amount of Ca^2+^ and Mg^2+^ ions), traditional gels often undergo rapid dehydration collapse or network disintegration due to the strong “salt-sensitive” effect, resulting in the shortening of the effective period of water plugging, which makes it difficult to meet the needs of deep and long-term profile control in the oil field.

Stimuli-responsive hydrogels exhibit large and often rapid changes in volume in response to changes in environmental conditions such as temperature [9], pH [10], ionic strength [11], and magnetic fields [12]. Thanks to their tunable physicochemical properties, these materials have been widely investigated for potential applications in sensing platforms, tissue engineering, controlled drug delivery, and wound management [13,14,15]. In order to overcome the limitations of traditional gels in profile control in low-permeability fractured reservoirs, intelligent responsive hydrogels that use the reservoir’s own temperature field or pH gradient to trigger state changes have become a research frontier. This type of gel maintains a low-viscosity solution state during the injection stage to facilitate deep penetration. After reaching the target fracture, a sol–gel transition or volume phase transition occurs in situ, building a high-strength sealing barrier. At present, most temperature-sensitive gels are represented by poly(N-isopropylacrylamide) (PNIPAM), which achieves thermally induced phase transition by adjusting the LCST [16,17]. pH-responsive gels are mainly based on the protonation-deprotonation dynamic equilibrium process of amino groups or carboxyl groups on the polymer chain, driving water expansion or cross-linking solidification [18,19].

In recent years, hyperbranched polymers have received considerable interest as functional polymeric materials because of their distinctive three-dimensional globular architecture, rich terminal functional groups, relatively low solution viscosity, and excellent molecular design. Liang [20] et al. synthesized hyperbranched poly (amino esters) containing terminal acrylate groups through a Michael addition reaction using acrylate and primary and secondary amines as monomers. The PEG chains were subsequently introduced onto the periphery of the hyperbranched poly (amino ester), and doxorubicin was coupled to the internal hydroxyl group through an acid-soluble hydrazine bond, thereby obtaining an anticancer drug-loaded hyperbranched polymer. The results showed that the obtained hyperbranched polymer exhibited good single-molecule stability and intracellular pH-responsive drug release behavior. Wang [21] prepared blended films by mixing PVA with water-soluble hyperbranched polyester (HBPE) using the solution casting method. The results showed that the hydroxyl groups of HBPE could form a hydrogen-bond-based cross-linking network with the hydroxyl groups of PVA. This, in turn, effectively improved the mechanical properties, water absorption, thermal stability, etc.

Polyethylene glycol diglycidyl ether (PEGDGE) is a bifunctional, highly water-soluble chemical that contains epoxy groups at both ends and can react with nucleophiles, including amino, mercapto, hydroxyl, and carboxyl groups [22]. Because of its good water solubility, good biocompatibility, and chemical stability, it is often used as an ideal crosslinker for hydrogels and is widely used in the fields of membrane technology, pharmaceuticals, and biomaterials [23,24,25,26]. However, there are few reports on cross-linking broom-shaped hyperbranched polymers with PEGDGE to build hydrogel systems with reversible multiple environmental responses and high-strength sealing.

Herein, this study proposes a new multi-stimuli-responsive hydrogel construction strategy based on amino-terminated 1.0 G hyperbranched broom macromolecules and double epoxy-terminated PEGDGE. The thermal stability, viscoelastic rheological behavior, swelling-deswelling reversibility, and intrinsic mechanism of gels with different carbon chain lengths under different temperature, pH, and salinity environments were systematically studied. Finally, their plugging performance and erosion resistance to low-permeability fractures were evaluated through core flow simulation experiments. This research aims to provide a new deep profile control material with good injectability, long-term stability, and intelligent response behavior for high-salinity, low-permeability fractured reservoirs.

## 2. Results and Discussion

### 2.1. Chemical Structure of Hyperbranched Macromolecules 1.0 G

**Figure 1:**
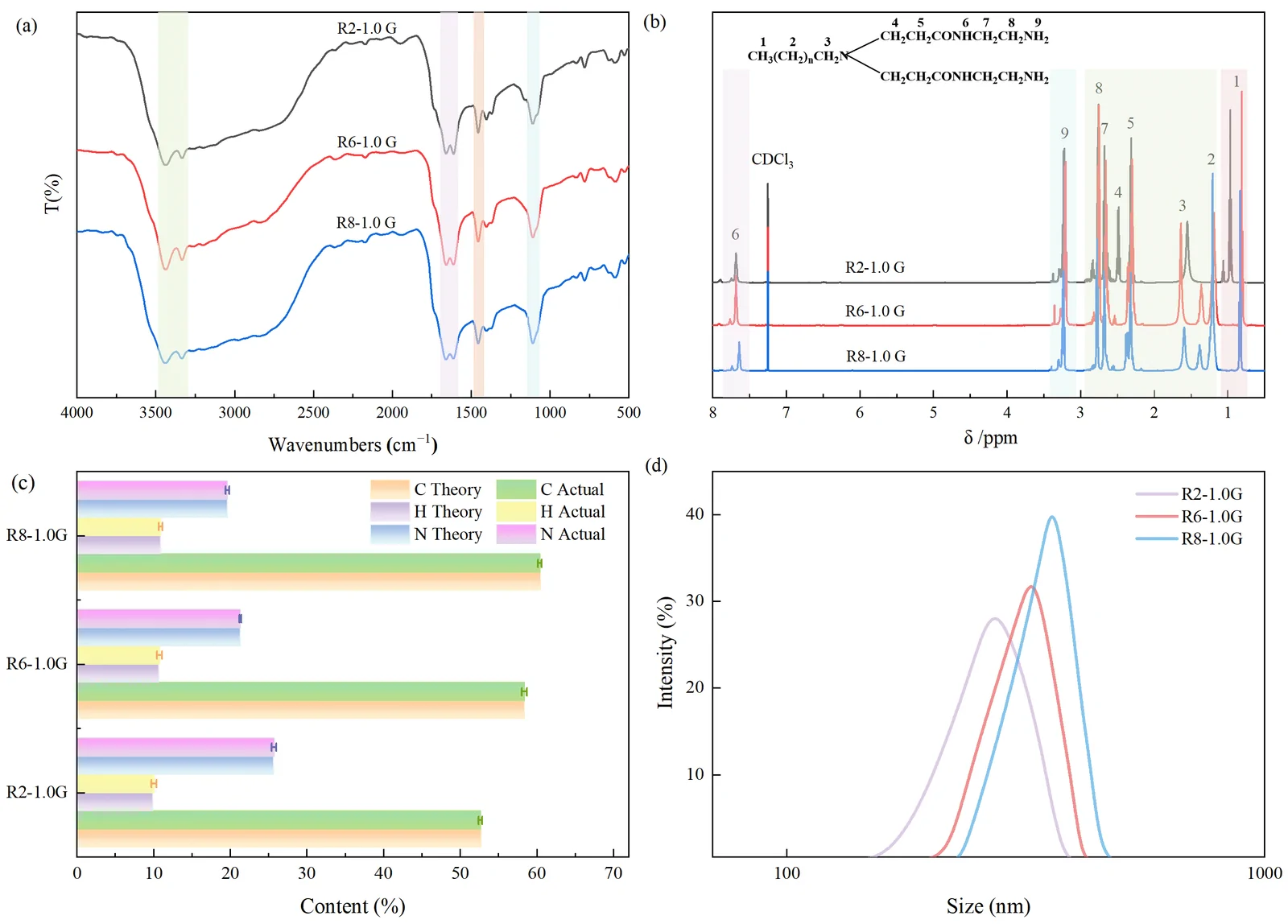
(**a**) FT-IR; (**b**) ^1^H NMR; (**c**) EA results and (**d**) particle size distribution of 1.0 G hyperbranched macromolecules.

Salient FTIR spectra are shown in Figure 1a. The infrared spectra of the products with three different alkyl cores exhibit a high degree of consistency. Strong absorption peaks appear at wavenumbers of 3438 cm^−1^ and 3300 cm^−1^, attributed to N-H and O-H stretching vibrations; the characteristic peak at 1455 cm^−1^ is attributed to C-H stretching vibrations on the alkyl chain. More importantly, significant absorption peaks were observed at 1638 cm^−1^ and 1550 cm^−1^, which can be attributed to the amide I band (C=O stretching vibration) and amide II band (N-H bending vibration coupled with C-N stretching vibration) in the amide group, respectively [27]. The appearance of these characteristic peaks indicates that after Michael addition, the 0.5 G ester intermediate was successfully amidated with EDA, forming the skeleton structure of the 1.0 G product. Figure 1b shows the ^1^H NMR spectrum of the 1.0 G polymer. The methyl proton signal appears at 0.83 ppm (H-1), and the methylene proton signals appear at 1.21 ppm (H-2), 1.59 ppm (H-3), 2.56 ppm (H-4), 2.32 ppm (H-5), 2.68 ppm (H-7), and 2.78 ppm (H-8), respectively. Furthermore, the resonance observed at 3.23 ppm was assigned to the H-9 protons associated with the terminal primary amine groups, whereas the peak at 7.64 ppm was attributed to the amide proton H-6 [28]. The presence of these characteristic resonances confirms the formation of amide functionalities and primary amine end groups, and each proton signal peak corresponds to the chemical structure. Therefore, ^1^H NMR and FT-IR analyses confirmed that a series of target products (C2-1.0 G, C6-1.0 G, C8-1.0 G) had been successfully synthesized as expected and possessed good structural regularity. The chemical composition of the series of 1.0 G hyperbranched macromolecules was characterized by elemental analysis (EA), and the corresponding results are presented in Figure 1c. The experimentally determined mass fractions of carbon (C), hydrogen (H), and nitrogen (N) for all samples closely agree with their calculated theoretical values, with very little error, indicating that this divergent synthesis strategy provides extremely high reaction conversion. With the increase in the carbon chain length of the alkyl core, the proportion of hydrophobic hydrocarbons in the molecule increases, the measured content of the C element in the product increases step by step, and the proportion of the N element decreases relatively. This result further confirmed at the elemental level that the target hyperbranched skeleton structure was successfully constructed as expected. The hydrodynamic size distributions of this group of chemicals in aqueous solution were described using dynamic light scattering (DLS), as illustrated in Figure 1d. C2, C6, and C8 were each tested at a concentration of 500 mg·mL^−1^ in deionized water at 25 °C. The DLS measurements were carried out at concentrations significantly higher than their independently estimated critical micelle concentrations (CMCs), which were roughly 0.20 mol·L^−1^ (54.6 mg·mL^−1^), 45 mmol·L^−1^ (14.8 mg·mL^−1^), and 12 mmol·L^−1^ (4.3 mg·mL^−1^), respectively. The findings show that the carbon-chain length had a significant impact on aggregate size; all three compounds formed supramolecular aggregates with apparent hydrodynamic diameters of roughly 200–400 nm. These sizes indicate aggregate structures that form above the critical micelle concentration (CMC), rather than the primary aggregates that are present at the beginning of micellization.

As the number of carbon atoms in the alkyl chain increases from C2 to C8, the particle size distribution in aqueous solution shows a significant trend of shifting to larger sizes and narrowing. This shows that the elongation of the hydrophobic chain not only increases the size of the aggregate but also promotes an effective improvement in the particle size uniformity, and the distribution becomes more concentrated, which is mainly due to the stronger hydrophobic association caused by the long carbon chain [29].

### 2.2. Morphology and Chemical Composition of Gels

The hydrogel samples were pre-frozen and freeze-dried to remove unbound water, thus preserving the porous structure. The typical microstructure of hyperbranched hydrogels was characterized by scanning electron microscopy.

**Figure 2:**
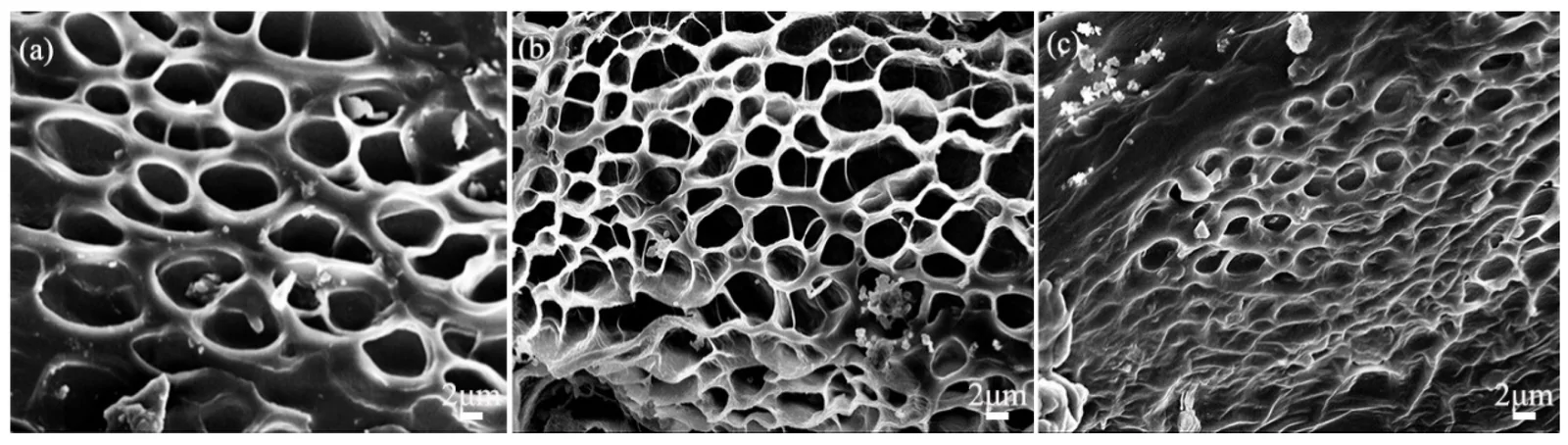
Scanning electron microscope images of (**a**) C2HG; (**b**) C6HG; (**c**) C8HG hydrogels.

Figure 2 shows the microstructures of C2HG, C6HG, and C8HG, respectively. The hydrogels exhibit a clear pore structure, and the addition of PEG contributes to the cross-linking density. This is because the PEG molecule is a flexible polymer chain, which changes the crystallization properties of the hydrogel to a certain extent, resulting in a large specific surface area and adsorption function [30]. This three-dimensional network structure of the hydrogel also improves water absorption. It can be seen that long-chain gels have a denser structure and fewer pores than short-chain gels [31]. When the molecular chain length is shorter, the pore size reaches approximately 13 μm, resulting in a hydrogel with a more open network structure.

**Figure 3:**
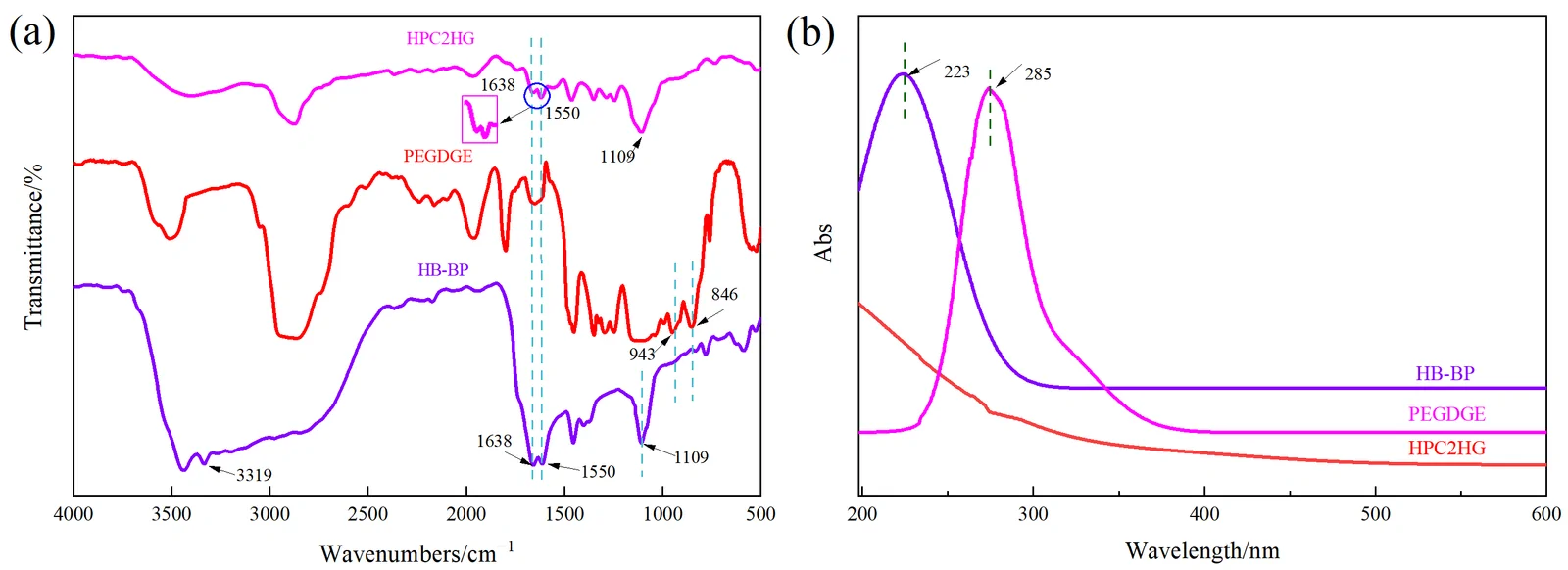
(**a**) FT-IR and (**b**) UV spectra of 1.0 G HBBP, PEGDGE, and HPHG.

The FTIR spectra of PEGDGE, the hyperbranched broom hydrogel (HPHG), and the 1.0 G hyperbranched broom polymer (HBBP) are displayed in Figure 3a. The spectra of PEGDGE and HPHG display broad absorption bands at 3200–3500 cm^−1^, assigned to the O–H stretching vibration. Absorption peaks are observed near 1638 cm^−1^ and 1550 cm^−1^ in both HBBP and HPHG spectra, which are attributed to the characteristic absorption bands of the amide moieties. The former absorption originates from the carbonyl stretching vibration, while the latter is attributed to the coupling of N–H bending and C–N stretching vibrations within the amide linkage. In addition, absorption peaks can be observed near 3319 cm^−1^ and near 1109 cm^−1^ in the spectrum of HBBP, which are the characteristic absorptions of amine groups and C-N bonds (secondary amine), respectively. There is a broad characteristic peak near 1150–1105 cm^−1^ in the PEGDGE spectrum, which is the characteristic absorption band of the -C-O-C group in aliphatic ethers [32]. In the spectrum of the hyperbranched broom-shaped hydrogel HPHG, the disappearance of the absorption bands at 1245, 943, and 846 cm^−1^ indicates that the epoxy groups were consumed through a ring-opening reaction with the amine groups [33]. The disappearance of the above characteristic peaks indicates that polyethylene glycol diglycidyl ether has been successfully grafted onto the hyperbranched broom-shaped polymer. Figure 3b shows the ultraviolet-visible absorption spectra of HBBP, PEGDGE, and HPHG. The results presented in Figure 3b indicate that HBBP exhibits an R band of n→π* transition from C=O at 223 nm. In contrast, the R band of the n→π* transition of the C=O bond in the ultraviolet spectrum of HPHG shows a red shift, and a characteristic absorption band appears at 285 nm [34], which further indicates that HBBP and PEGDGE cross-linked and a ring-opening reaction occurred, and the hydrogel was successfully prepared.

### 2.3. Thermal Stability

**Figure 4:**
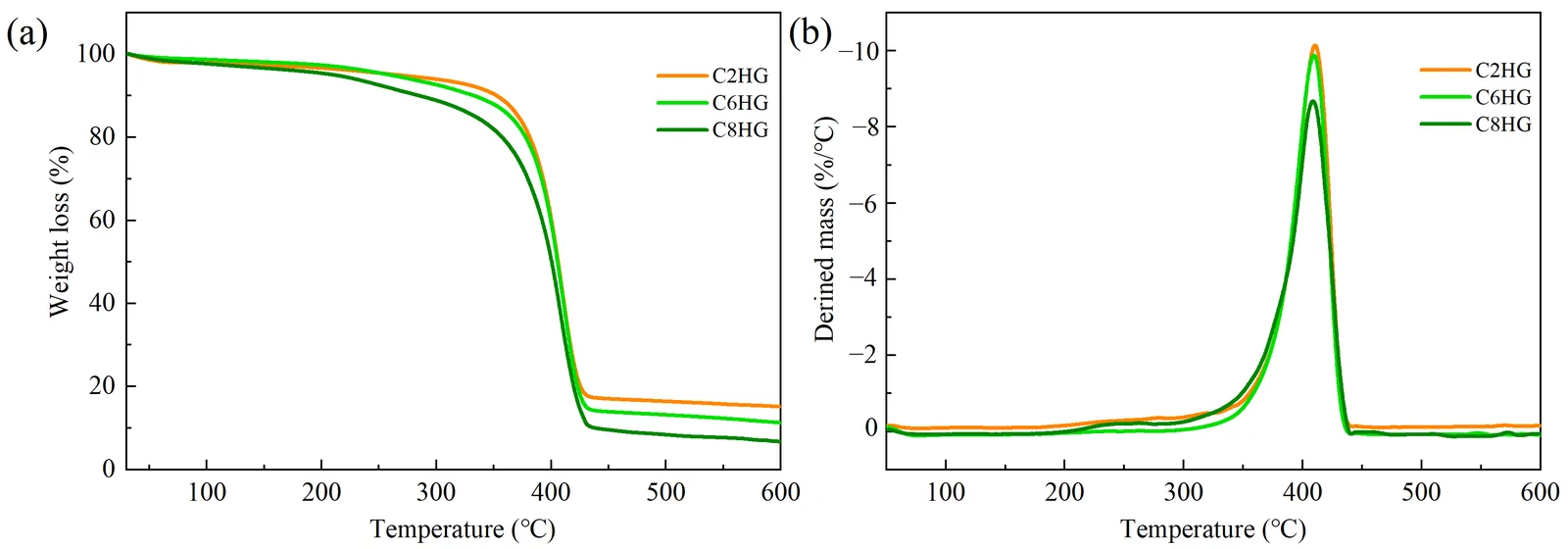
Thermal degradation profiles of the different hydrogels: (**a**) TG curves and (**b**) DTG curves.

The thermogravimetric analysis (TGA) curves and derivative thermogravimetric (DTG) curves of the series of copolymer hydrogels (C2HG, C6HG, and C8HG) under a nitrogen atmosphere are shown in Figure 4a,b. The thermal degradation behavior of all samples can be divided into three main stages, and key parameters are summarized in Table 1 for clearer comparison. The evaporation of physically adsorbed water in the three-dimensional network and residual polar solvents or other low-molecular-weight compounds were the main causes of the slight mass loss observed in all samples between room temperature and 200 °C, suggesting that the gel system has good hydrophilicity. The curves dropped sharply between 300 and 450 °C, and the weight loss rate reached more than 80%. The severe mass loss at this stage corresponds to the thermal rupture and depolymerization of carbon-carbon bonds, carbon-nitrogen bonds, and ether bonds in the polymer backbone and linear PEG segments in the hydrogel. The residual mass stabilizes and the mass loss rate slows considerably after 450 °C. A comparison of the residual carbon contents among the three samples reveals that, with increasing alkyl chain length from C2 to C8, the residual mass fraction shows a slight decreasing trend. This is because longer linear alkyl groups are more likely to form volatile short-chain hydrocarbons or small-molecule gases and escape during high-temperature pyrolysis, resulting in a relative reduction in the residual carbonized skeleton.

A single sharp peak corresponding to the maximum weight loss rate is observed at approximately 400 °C, mainly owing to the dominant polyether segments in the system and the thermal decomposition and dehydration of amide/ester bonds in the hyperbranched skeleton [35]. The T_50%_ for the hydrogels are 406.1 °C, 405.6 °C, and 400.4 °C, respectively. The decomposition temperatures of C6HG and C8HG are slightly lower than that of C2HG, which can be attributed to the slight decrease in thermal stability caused by the increase in the proportion of aliphatic carbon-hydrogen bonds in the long alkyl segment [36]. Despite minor differences, TGA results showed that the series of hyperbranched broom-shaped hydrogels all exhibited excellent thermal stability.

### 2.4. DSC Measurement

**Figure 5:**
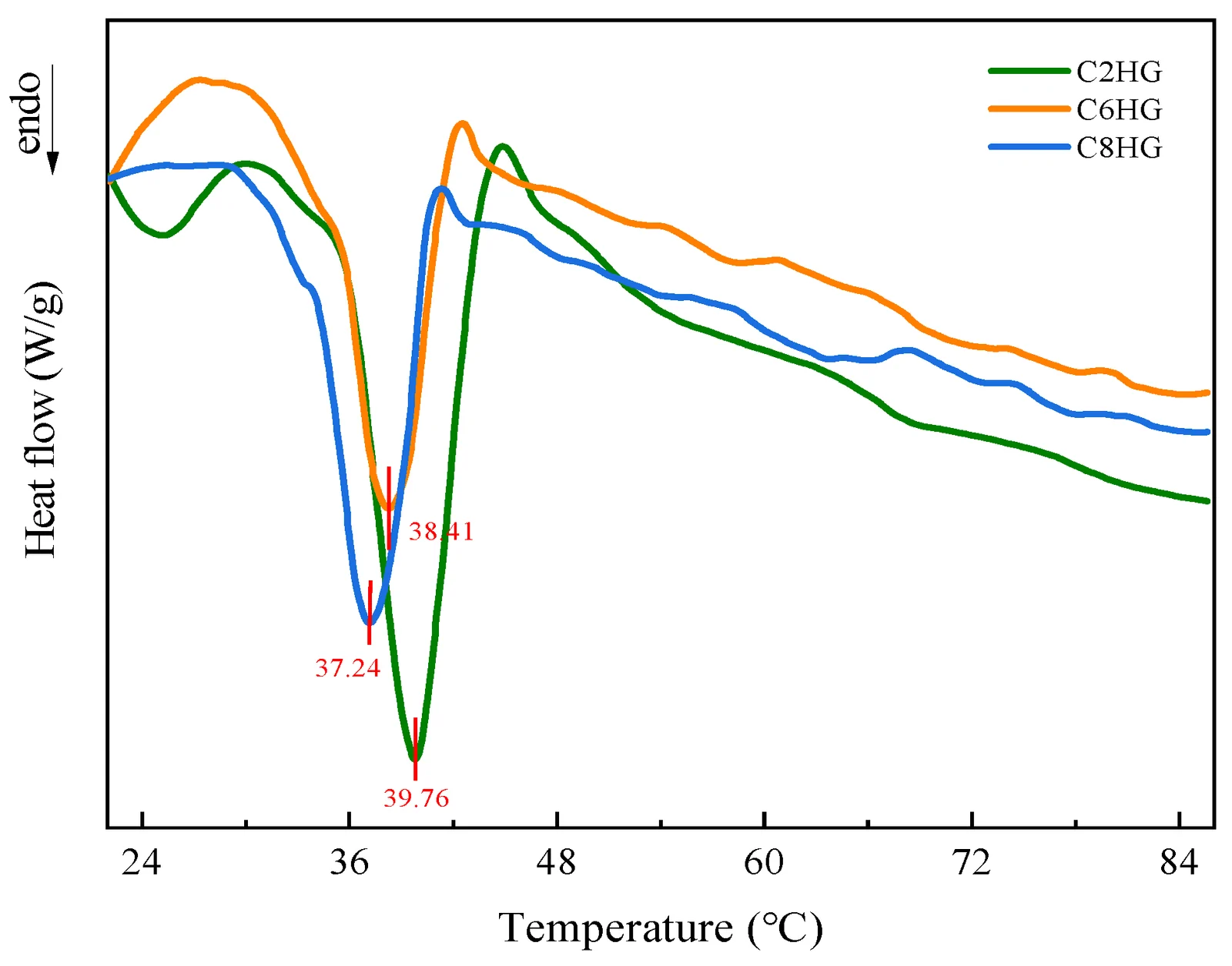
DSC heating curves of C2HG, C6HG, and C8HG.

The LCST of the hydrogels was characterized by differential scanning calorimetry (DSC). It can be observed from Figure 5 that hydrogels with different chain lengths have an obvious endothermic peak in the range of 37.3–39.8 °C, which usually corresponds to the volume phase transition that occurs when the hydrophilic/hydrophobic balance in the hydrogel network is broken, i.e., the LCST value [37]. With the increase in carbon chain length in the system, the peak shows a regular trend of shifting to lower temperatures. This can be explained by the growth of the alkyl chain and the significant enhancement of hydrophobic association between molecules. The stronger hydrophobic effect destroys the more ordered “hydration layer” around the polymer segments, allowing the system to require only lower thermal energy (lower temperature) to drive the separation of water molecules inside the hydrogel and the network to undergo hydrophobic collapse and dehydration, which causes the endothermic phase transition temperature to move towards lower temperatures. At the same time, the results show that by selecting starting cores with different carbon chain lengths, the temperature-sensitive phase transition behavior of hydrogels can be effectively and controllably fine-tuned.

### 2.5. Rheological Properties

**Figure 6:**
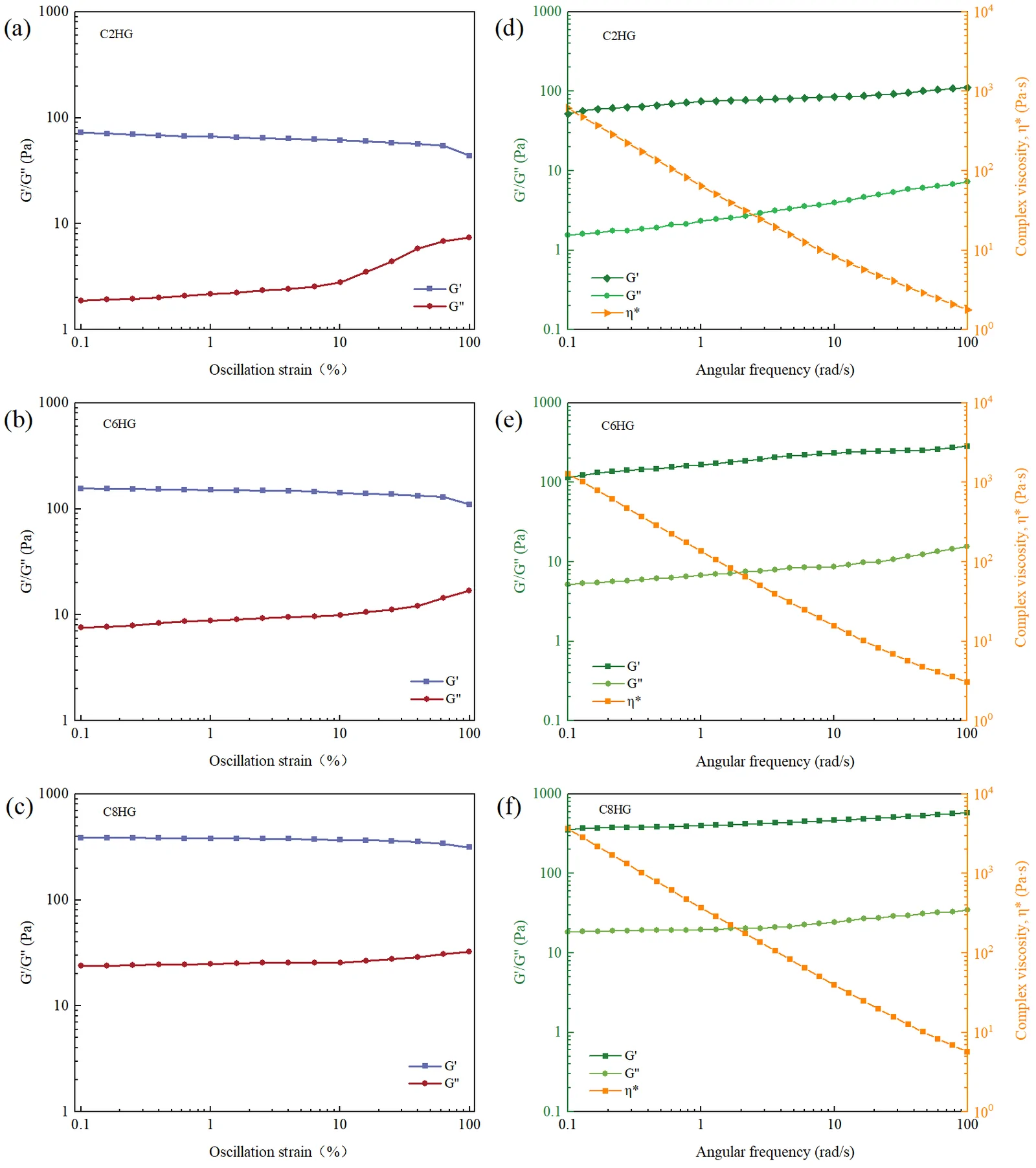
Rheological behavior of C2HG, C6HG, and C8HG: oscillatory strain sweep (**a**–**c**), angular frequency sweep, and complex viscosity curve (**d**–**f**).

Figure 6 shows the rheological behavior of copolymer hydrogels (C2HG, C6HG, and C8HG) with different backbone chain lengths, including oscillatory strain scans (Figure 6a–c) and angular frequency scans and complex viscosity curves (Figure 6d–f). Figure 6a–c illustrates that, within the tested strain range (0.1–100%), the storage modulus (G′) of each gel sample is always much greater than the loss modulus (G″), indicating that all systems present a typical elastic-dominated state before being destroyed [38,39]. In the low strain range of 0.1% to about 20–30%, both G′ and G″ maintain relatively stable plateau regions, defined as the linear viscoelastic region of the hydrogel. When the oscillating strain exceeds the critical yield strain (about 30–40%), G′ shows a significant steep drop, while G″ gradually increases, indicating that the physical cross-linking points within the three-dimensional network of the gel have been destroyed and the system has yielded and entered a nonlinear viscoelastic flow state. In addition, as the carbon chain skeleton length increases (from C2 to C8 alkyl chains), the plateau modulus of the hydrogel increases significantly. This phenomenon is highly consistent with the aforementioned particle size distribution and hydrophobic association mechanism: the stronger hydrophobic association effect brought by the long carbon chain skeleton and the enhanced physical entanglement between polymer chains [32] promote a significant increase in the cross-linking density within the gel network, which in turn greatly improves the overall mechanical strength of the system.

The dynamic viscoelastic response of the hydrogels was further evaluated by angular frequency scanning (Figure 6d–f). In the full frequency range of 0.1 to 100 rad/s, the G′ of all samples is always higher than G″, and both moduli show a slight increasing trend with the increase in frequency. The high-frequency rheological data reveal a predominantly elastic response, indicating that the three-dimensional hydrogel network maintains its structural integrity and exhibits a high capacity for elastic energy storage. Nevertheless, a minor viscous contribution remains detectable under rapid deformation, which may be associated with the pressure-driven migration and expulsion of water through the interconnected pore channels. This fluid-dependent response is analogous to the poroelastic behavior of cartilage, in which mechanical loading induces fluid transport and consequently contributes to time-dependent deformation [40].

In addition, looking at the right auxiliary longitudinal axis, it can be found that the complex viscosity (η*) of the hydrogel decreases sharply with the increase in angular frequency, and the curve almost conforms to the shear-thinning behavior. When the angular frequency increased to 100 rad/s, the viscosity of the three samples declined sharply to about 10 Pa·s. This remarkable shear-thinning behavior of non-Newtonian fluids is of great significance to practical engineering applications. It means that the viscosity of the hydrogel system is greatly reduced when pumped and injected into the pores of the formation (at high shear rates), providing excellent injectability and allowing smooth passage through the tiny pore throats of low-permeability formations; once it enters the target fracture area, the flow rate slows down (low shear rate), and the system can quickly return to high viscosity and establish a high-strength gel network.

Comprehensive strain and frequency scanning results show that the hydrogels possess a high storage modulus and low-frequency viscosity due to their strong hydrophobic association network structure. This excellent viscoelastic mechanical property provides a solid structural foundation for withstanding high-pressure fluid erosion in actual fracture sealing. The conclusion of gel strength enhancement drawn from rheological analysis is consistent with the extremely high plugging rate of 99.9% and high breakthrough pressure in subsequent core plugging experiments. To sum up, the PEG-terminated 1.0 G series copolymer hydrogels possess injectable shear-thinning properties, high-viscosity recovery in deep reservoirs, and high mechanical strength, making them promising for deep liquid flow diversion and long-term profile control and water shutoff in low-permeability fractured reservoirs.

### 2.6. Swelling Properties

Swelling is one of the key characteristics of hydrogels. As the hydrogel swells, the solvent penetrates the polymer, causing volume expansion and stretching the molecular chains between network crosslinks into three-dimensional space. The stretched network generates an elastic retractive force that restricts further swelling. As swelling proceeds, this retractive force increases while the swelling driving force decreases Finally, these two forces reach a balance, namely, swelling equilibrium. The effects of the following factors on the equilibrium swelling ratio (ESR) were investigated: (1) 1.0 G hyperbranched molecules with different chain lengths, (2) temperature, (3) pH, and (4) salinity.

**Figure 7:**
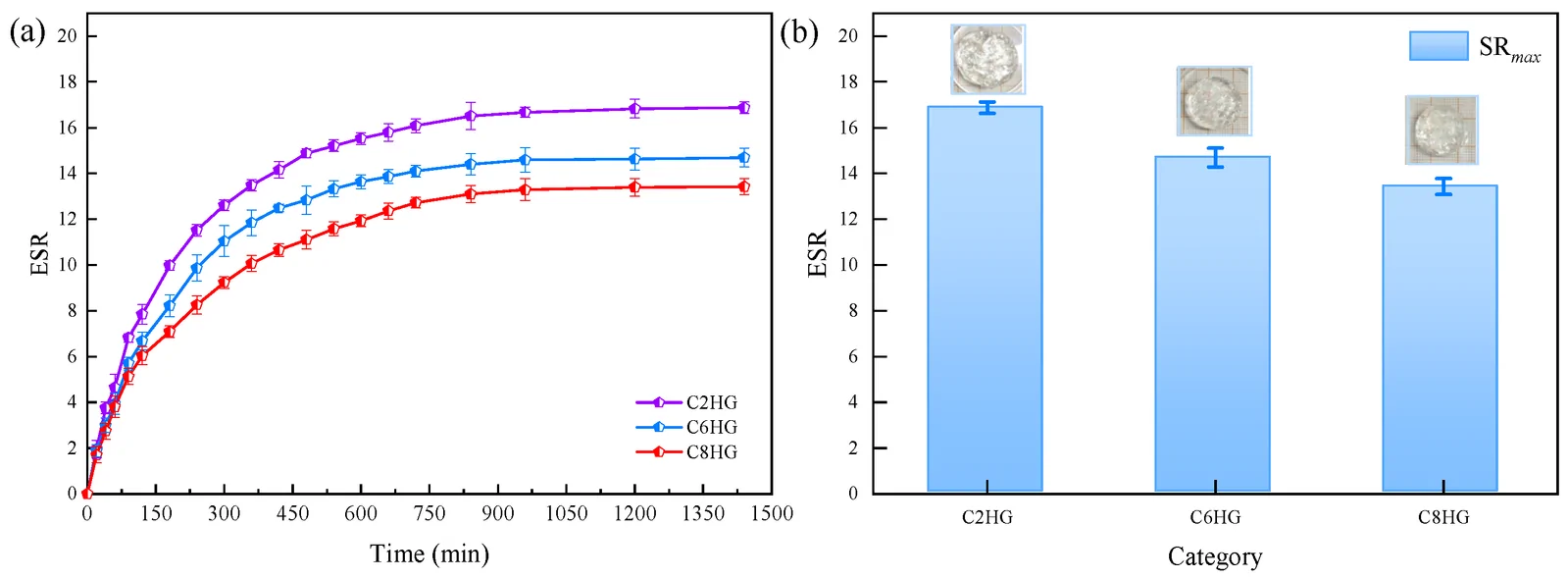
Curve of swelling ratio of different hydrogels versus time: (**a**) dynamic swelling; (**b**) equilibrium swelling.

As shown in Figure 7a, three groups of hydrogel samples gradually expanded with time and reached swelling equilibrium within 900 min, with equilibrium swelling ratios of 16.8, 14.7, and 13.4, respectively (Figure 7b). These hydrogels exhibited relatively high equilibrium swelling ratios [41]. Among them, PEGDGE, as a highly hydrophilic polymer [42], has a large number of hydroxyl groups in its molecular chain, which can form hydrogen bond interactions with water molecules and has a strong attraction to water molecules. When added to hydrogels, its hydrophilicity helps attract water molecules into the hydrogel interior, extending the hydrogel network and forming pores, thus increasing the water content of the hydrogel [43].

The equilibrium swelling degree of hyperbranched broom-shaped hydrogels decreases gradually with the increase in molecular chain length. When the chain length is C2, the hydrogel reaches its maximum swelling. This is because the length of the molecular chain affects the cross-linking density. As the chain length increases, the cross-linking density increases, resulting in a narrower gap between the internal networks of the hydrogel, which hinders the movement of water molecules and increases the hydrophobic effect. Therefore, the amount of water absorbed by the hydrogel during swelling also decreases.

#### 2.6.1. Temperature-Responsive

**Figure 8:**
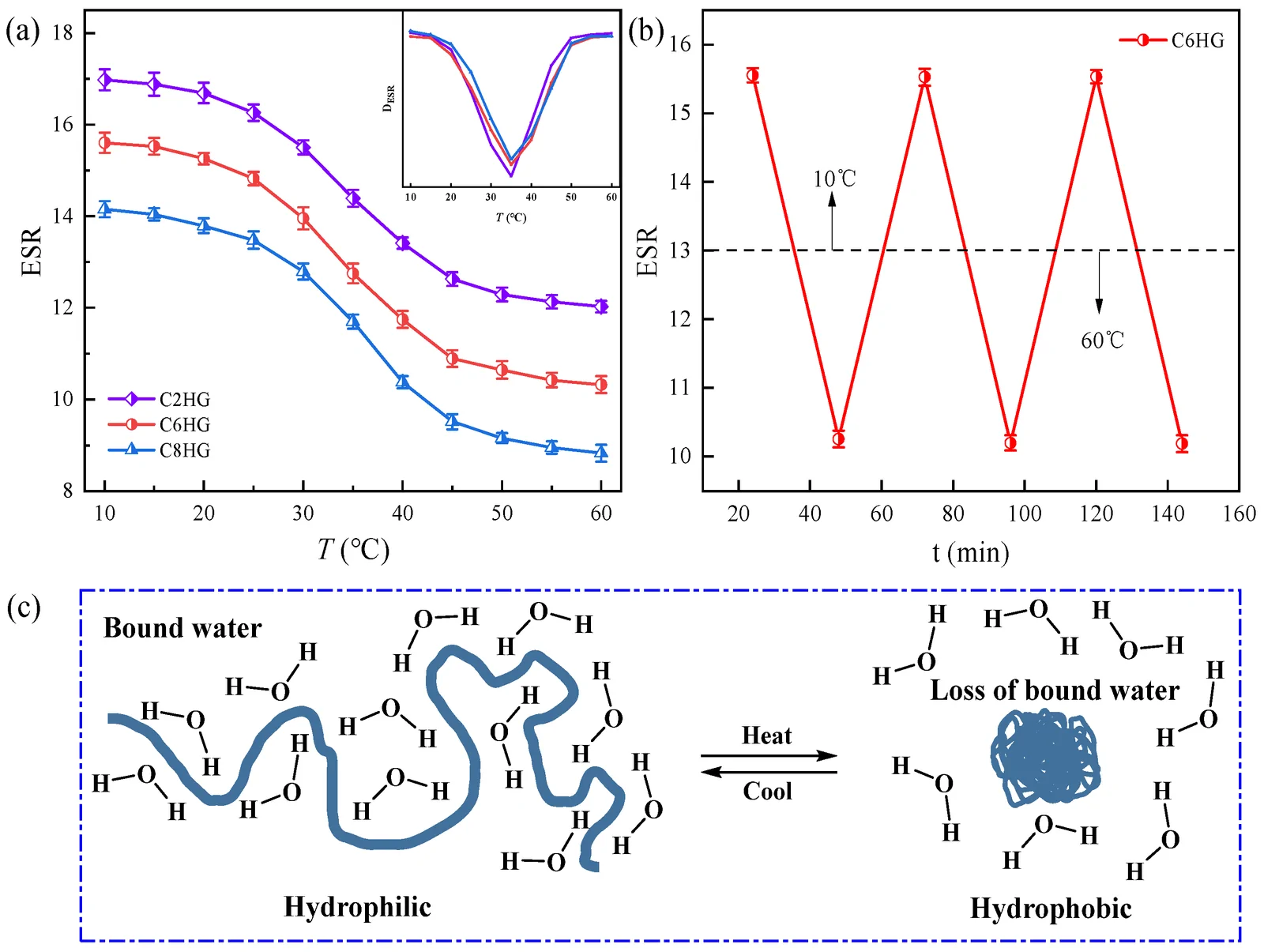
Temperature-sensitive response performance and mechanism of hydrogel: (**a**) ESR curve with temperature; (**b**) swelling–deswelling reversible cycle test; (**c**) schematic diagram of conformation transition.

Figure 8 shows the temperature-sensitive response properties and microscopic control mechanisms of the series of copolymer hydrogels. As can be observed in Figure 8a that at all tested temperatures, the ESR of the hydrogel decreases with the increase in carbon chain length, in the order of C2HG > C6HG > C8HG. This is mainly due to the fact that the long carbon chain skeleton gives the system a stronger hydrophobic association and promotes the formation of a denser physical cross-linked network inside the gel, thus limiting the penetration of water molecules into the polymer matrix. In terms of temperature sensitivity, with the increase in ambient temperature, the ESR of all samples showed a step-like decline trend similar to an “S-shaped” change [44,45]. At 10 °C, the swelling ratio is almost 17 times the original weight. When the temperature increased to around 37 °C, the gel underwent a violent volumetric phase transition, which corresponded to the DSC test results. Subsequently, as the temperature increased, the curve stabilized, and only a small degree of swelling occurred. This is because at low temperatures, hydrophilic groups in the molecular chain bind with water molecules through hydrogen bonds, causing the hydrogel to swell and the overall molecular chain to be in a relaxed state. The system entropy increases with increasing temperature, destroying the ordered hydration layer around the chain segments, resulting in the loss of bound water, weakening the hydrogen bond interaction, and causing the hydrophobic interaction between the polymer chains to dominate. As a result, water molecules are released from the hydrophobic aggregates, and the overall gel network collapses into a shrunken state, reducing the swelling ratio. See Figure 8c for a detailed schematic diagram of the mechanism.

Taking C6HG as a representative, ESR time-dependence tests under temperature cycles (10–60 °C) were carried out, and the corresponding results are presented in Figure 8b. During three complete temperature cycles, the hydrogel showed obvious reversible swelling-deswelling behavior. This response characteristic ensures that the gel can maintain the integrity of its physical structure in the face of fluctuations in formation temperature in low-permeability reservoirs and has great engineering potential in deep profile control and water shutoff applications.

#### 2.6.2. pH-Responsive

**Figure 9:**
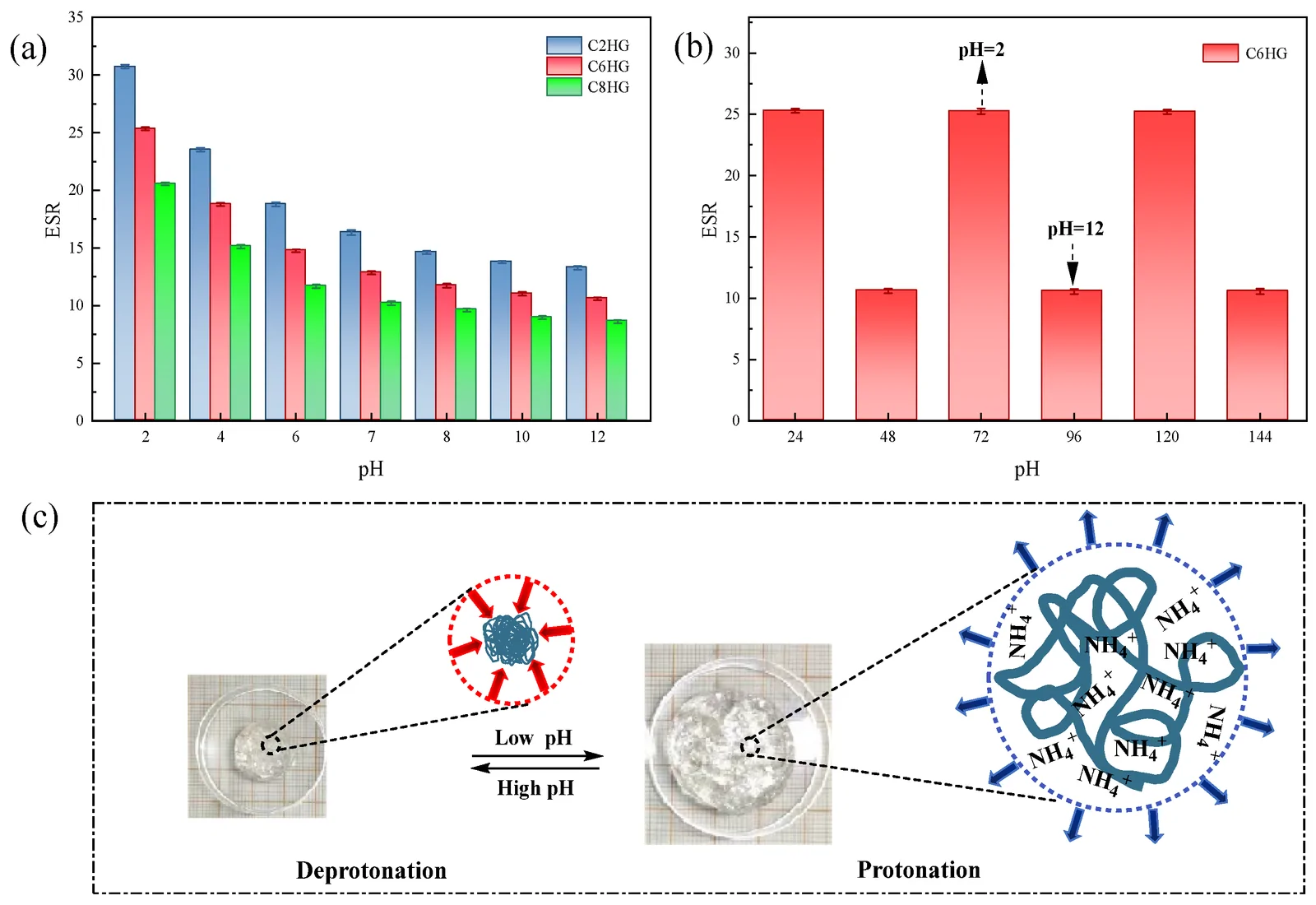
pH response behavior of hydrogels: (**a**) ESR changes with pH value; (**b**) alternating cycle test of C6HG hydrogel between pH 2 and pH 12; (**c**) macroscopic photographs and schematic diagram of the microscopic molecular mechanism of pH response.

Figure 9 shows the swelling properties, response reversibility, and intrinsic mechanism of the series of copolymer hydrogels under different pH conditions. As can be observed in Figure 9a that the ESR of the three hydrogels shows significant differences under different pH environments. Overall, when the test environment was acidic (pH = 2–6), the system had a high ESR value. The maximum swelling ratio of the short-chain hydrogel C2HG at pH = 2 was as high as 30.7, while C6HG and C8HG dropped to 25.3 and 20.5, respectively. As the pH value increased towards the alkaline range (pH = 8–12), the ESR of all hydrogels showed a step-by-step decline trend and tended to a stable low-swelling state at pH > 10. At the same time, under all pH conditions, the swelling order of C2HG > C6HG > C8HG is maintained, which once again confirms that the hydrophobic association caused by long carbon chains will offset part of the swelling ability caused by electrostatic repulsion. This shows that these hydrogels exhibit significant differences in swelling degrees under acidic and alkaline conditions and possess typical pH response characteristics.

To investigate the structural stability of the gel network under extreme acid and alkali environments, cyclic swelling tests were performed between pH 2 and 12, taking C6HG hydrogel as a representative. As shown in Figure 9b, during three complete cycles, the ESR value of the gel quickly recovered to around 25 at pH = 2 and shrank to around 10 at pH = 12. After multiple alternating stimuli of strong acid and strong base, the swelling and deswelling values of the gel fluctuated very little. This result strongly proves that the copolymer hydrogel has extremely excellent pH response reversibility and chemical structure stability and can adapt to the complex acid-base fluid environment that may occur in actual reservoir conditions while maintaining the integrity of the network structure without hydrolysis damage.

Combining the macroscopic photographs in Figure 9c with the micromolecular model, the pH response behavior of the hydrogel can be deeply explained. In an acidic environment, the primary amino group (-NH_2_) at the terminal group of the 1.0 G hyperbranched skeleton is protonated and converted to cationic -NH_3_^+^, and the charge density increases. The electrostatic repulsive force between the same charges enhances the stretching of polymer segments. At the same time, the ionic groups greatly enhance the hydrophilicity of the network, causing a large amount of water molecules to pour in, and the gel is in a swollen state. The macroscopic manifestation is that the gel volume expands and transparency increases. Conversely, in an alkaline environment (high pH), -NH_3_^+^ in the system undergoes deprotonation and returns to -NH_2_, and the electrostatic repulsion force quickly disappears. At this time, the inherent hydrophobic association between the long carbon chain skeletons reoccupies the dominant position, forcing the polymer network to shrink sharply and expel bound water [46,47], which results in obvious volume shrinkage and a low ESR.

#### 2.6.3. Salinity-Responsive

**Figure 10:**
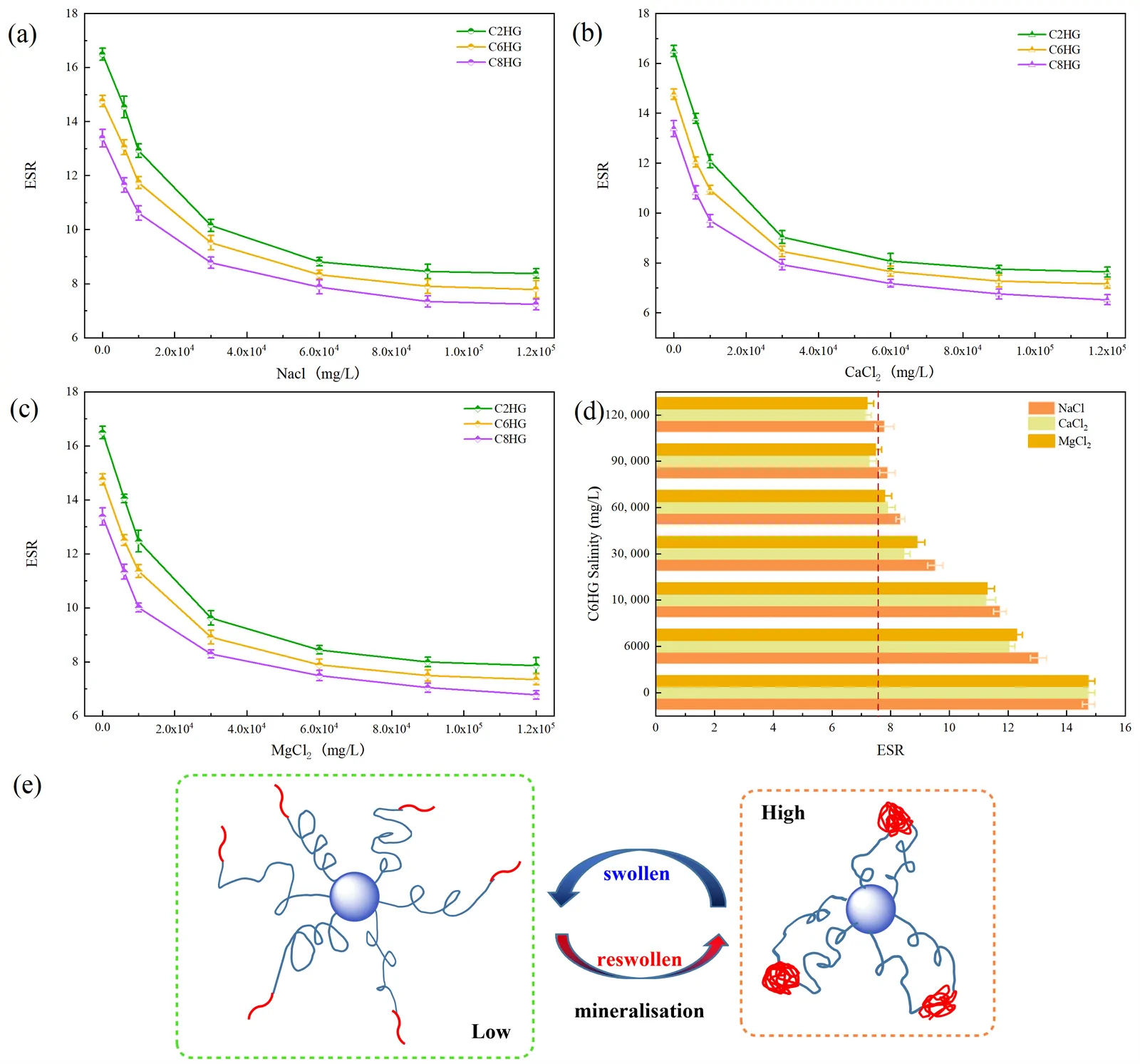
Salinity response and salt-tolerance test of hydrogels: (**a**–**c**) changes in ESR in NaCl (**a**), CaCl_2_ (**b**), and MgCl_2_ (**c**) solutions; (**d**) ESR comparison chart of C6HG at different salinities; (**e**) schematic diagram of the mechanism of stretching and hydrophobic association.

Figure 10 explores the effect of environmental salinity on the swelling behavior of the series of copolymer hydrogels and their micro-control mechanism. As shown in Figure 10a–c, the changes in ESR of hydrogels with salinity in three different salt solutions, NaCl, CaCl_2_, and MgCl_2_, were investigated. Overall, the ESR of the three gels showed an “L-shaped” change trend that first dropped sharply and then tended to level off with the increase in salinity. When the external salinity increased from 0 to about 40,000 mg/L, the gel volume shrank violently; when it continued to increase to a highly mineralized environment of 120,000 mg/L, the ESR decline rate significantly slowed down and reached swelling equilibrium. This is mainly due to the charge screening effect and the salting-out effect. As the concentration of counterions increases in the solution, the electrostatic repulsion force generated by protonated amino groups (-NH_3_^+^) in the hydrogel network is effectively screened by a large number of inorganic salt ions, resulting in weakened electrostatic repulsion between polymer segments.

Meanwhile, the hydration of high concentrations of salt ions strips away the hydration layer around the polymer chains, greatly weakening the hydrophilic interaction, and allowing the originally suppressed hydrophobic association to dominate again [48], driving the cross-linked network to shrink violently and drain water. In addition, across different salinity ranges, the ESR of the three hydrogels always maintains the rule of C2HG > C6HG > C8HG, which is consistent with the previous hydrophobic interaction conclusion: the long carbon chain skeleton (C8HG) has a stronger hydrophobic aggregation tendency. Under the synergistic effect of the salting-out effect, its three-dimensional network is more restricted [49], and its swelling degree is lower. Figure 10d takes C6HG as an example to visually compare the differential effects of the metal cation types of NaCl, CaCl_2_, and MgCl_2_ on gel swelling under the same mass concentration gradient. Observations show that with the increase in salinity, the ESR decrease caused by CaCl_2_ and MgCl_2_ is slightly greater than that caused by NaCl. This is usually because divalent metal cations (Ca^2+^, Mg^2+^) have higher ionic charge density and stronger hydration ability. They can not only shield electrostatic repulsion more efficiently but may also generate additional physical cross-linking with amino and amide groups in the gel network through coordination, thereby further strengthening the shrinkage of the gel network. This shows that the hydrogel can still maintain stable response behavior in highly mineralized formation water dominated by divalent ions.

Figure 10e reveals the intrinsic mechanism of the salinity-responsive behavior of hydrogels using a molecular conformation model. At low salinity, the protonated amino groups on the polymer chain segments carry a large amount of positive charge. The strong electrostatic repulsion force keeps the polymer chain in a completely stretched conformation. A large number of water molecules enter, and the gel network is in a swollen state. When the environment changes to a high-salinity environment, the charge-shielding effect of the counterions eliminates electrostatic repulsion and promotes strong hydrophobic association and collapse of the hydrophobic segments on the polymer skeleton. The gel quickly discharges water, showing a deswollen/shrunken state. The stretching and contraction of this conformation are highly reversible when salinity alternates.

Comprehensive salt resistance test results show that this series of hydrogels still maintains its inherent three-dimensional network structure in a highly mineralized environment of up to 120,000 mg/L and has excellent conformational reversibility. It is particularly worth pointing out that in an environment with high salinity (especially formation water containing high concentrations of Ca^2+^ and Mg^2+^), traditional polyacrylamide gels are prone to irreversible dehydration and collapse, producing a strong “salt-sensitive” effect, leading to polymer precipitation or network disintegration and plugging failure. However, the copolymer hydrogels prepared in this study still maintain structural stability and reversible stretching response under high salinity conditions due to their unique hyperbranched broom-like skeleton and reversible protonation/hydrophobic association balance. This has practical engineering significance for high-salinity and low-permeability fractured reservoirs in northwest China and provides a reliable material basis for realizing deep long-term plugging and fluid flow diversion.

### 2.7. Core Plugging Experiment

**Figure 11:**
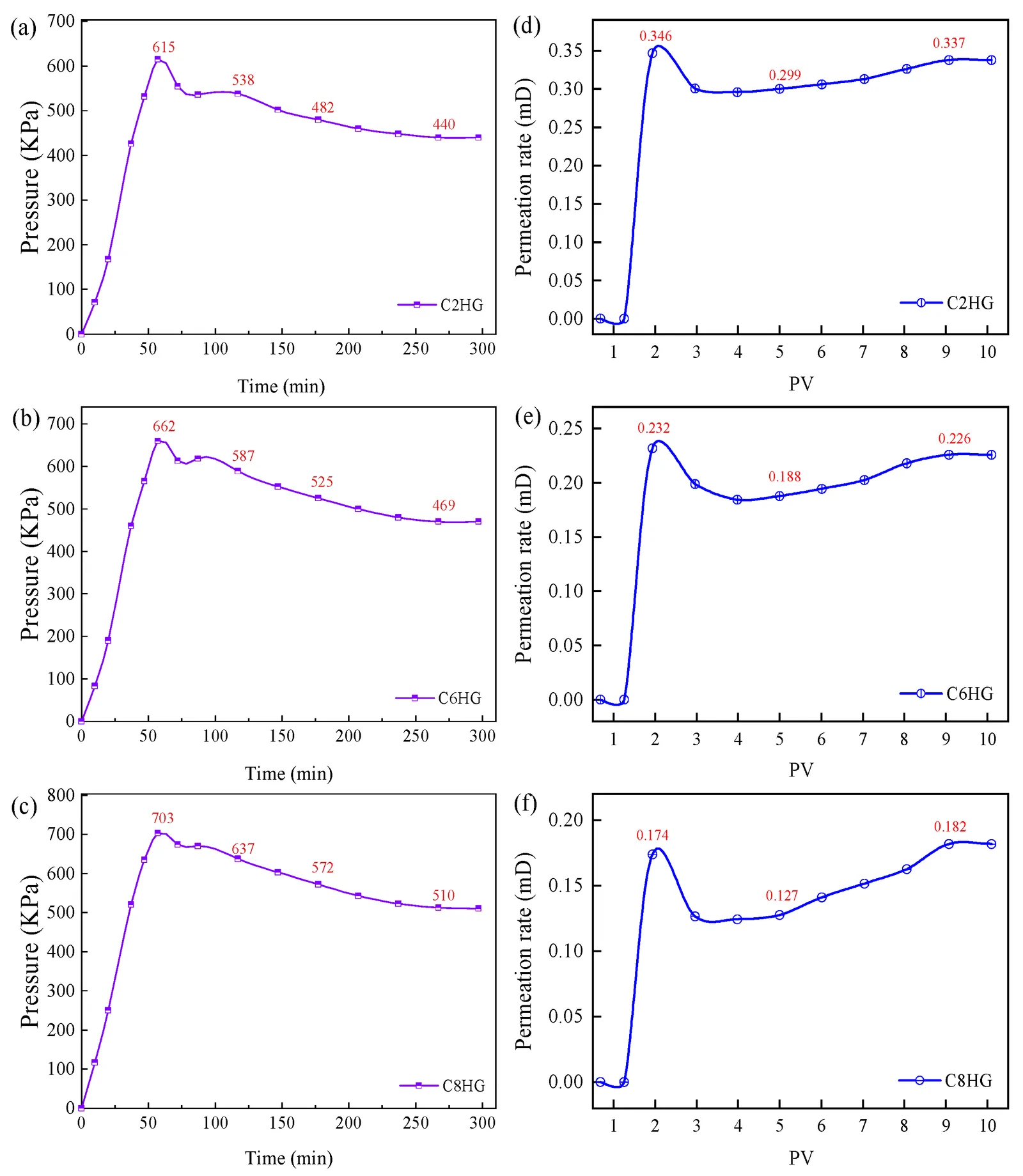
Core flow experimental plugging performance of C2HG, C6HG, and C8HG hydrogels: (**a**–**c**) injection pressure versus injection time; (**d**–**f**) permeability versus injected PV.

In order to verify the plugging performance of the series of copolymer hydrogels, core flow simulation experiments were carried out. Figure 11 shows the curves of injection pressure versus injection time (Figure 11a–c) and the curves of core permeability versus injected pore volume (PV) after plugging (Figure 11d–f). The detailed experimental results are summarized in Table 2. As illustrated in Figure 11a–c, the injection pressure rises rapidly with increasing injection time. After 50 min of injection, the pressure increased rapidly to break through the core, reaching 600–700 kPa. After breaking through the peak pressure, the pressure curve showed a slight downward trend and then entered a stable high-pressure plateau period. The increase in initial pressure can be attributed to the absence of a water transport path within the gel, while the sudden drop in differential pressure is due to the formation of wormholes or new permeation paths. This phenomenon is similar to the work reported by Jiang et al. [50]. The above changes show that the gel establishes a stable physical plugging barrier in the rock core.

Erosion resistance is an important indicator for evaluating the water plugging effect and validity period, as shown in Figure 11d–f. In the initial stage of simulated water injection, the permeability of the core was extremely low (close to 0 mD), indicating that the injected gel system effectively blocked the fluid channel. As the injection amount increases, the gel migrates and breaks through in the core, causing the permeability curve to peak at approximately 2.0 PV injection. After that, the permeability showed a brief trough of decline, then increased slightly with the slow passage of residual gel and gradually leveled off. This shows that even after experiencing a high-pressure breakthrough, the gel network still has extremely strong viscoelasticity and self-healing properties, which can effectively inhibit the advancement of the aqueous phase, and the core water plugging rate can be maintained above 99.9%, showing excellent erosion resistance and effective plugging potential. This confirms that the PEG-terminated hyperbranched copolymer hydrogels have excellent application prospects in the field of deep conformance control and water shutoff in low-permeability fractured reservoirs.

## 3. Conclusions

Here, we report the successful synthesis of hydrogels C2HG, C6HG, and C8HG with well-defined structures using linear alkylamines (C2, C6, and C8) as building blocks and bridging with α,ω-diepoxy-terminated polyethylene glycol by a one-pot method. The resulting hydrogels exhibit excellent thermal stability below 300 °C and a three-dimensional interconnected porous network. The LCST shifts slightly to a lower temperature as the alkyl chain length increases because of increased hydrophobic association. These hydrogels respond reversibly to temperature, pH, and salinity, and their equilibrium swelling ratio can be successfully tuned by varying the alkyl chain length. Rheological analyses show that the gels are primarily elastic and exhibit notable shear-thinning behavior, which provides a mechanical foundation for their injectability. In core plugging experiments, a breakthrough pressure of nearly 700 kPa was attained, leading to a plugging efficiency of 99.9% and a significant decrease in water-phase permeability. In conclusion, these PEG-terminated hyperbranched copolymer hydrogels display excellent injectability, resilience to various environmental conditions, and sustained sealing efficacy, indicating significant potential for applications in deep profile management and water blockage in high-salinity, low-permeability fractured reservoirs.

## 4. Materials and Methods

### 4.1. Materials

Ethylamine (C_2_H_7_N), 1-hexylamine (C_6_H_15_N), and octylamine (C_8_H_19_N) were purchased from Sigma Co., Ltd. (Shanghai, China). Polyethylene glycol diglycidyl ether (PEGDGE, Mn = 1000 g/mol) was self-made [51]. Methanol (CH_3_OH) and ethylenediamine (EDA) were supplied by Tianjin Kemiou Chemical Reagent Co., Ltd. (Tianjin, China). Ammonium hydroxide (NH_4_OH), glacial acetic acid (CH_3_COOH), 95% ethanol, and methyl acrylate (C_4_H_6_O_2_) were obtained from Tianjin Damao Chemical Reagent Co., Ltd. (Tianjin, China). All reagents were of analytical grade and were used as received without additional purification. The solutions required for the experiments were prepared in ultrapure water.

### 4.2. Synthesis of Hyperbranched Broom Polymers (Cn-1.0 G)

**Figure 12:**
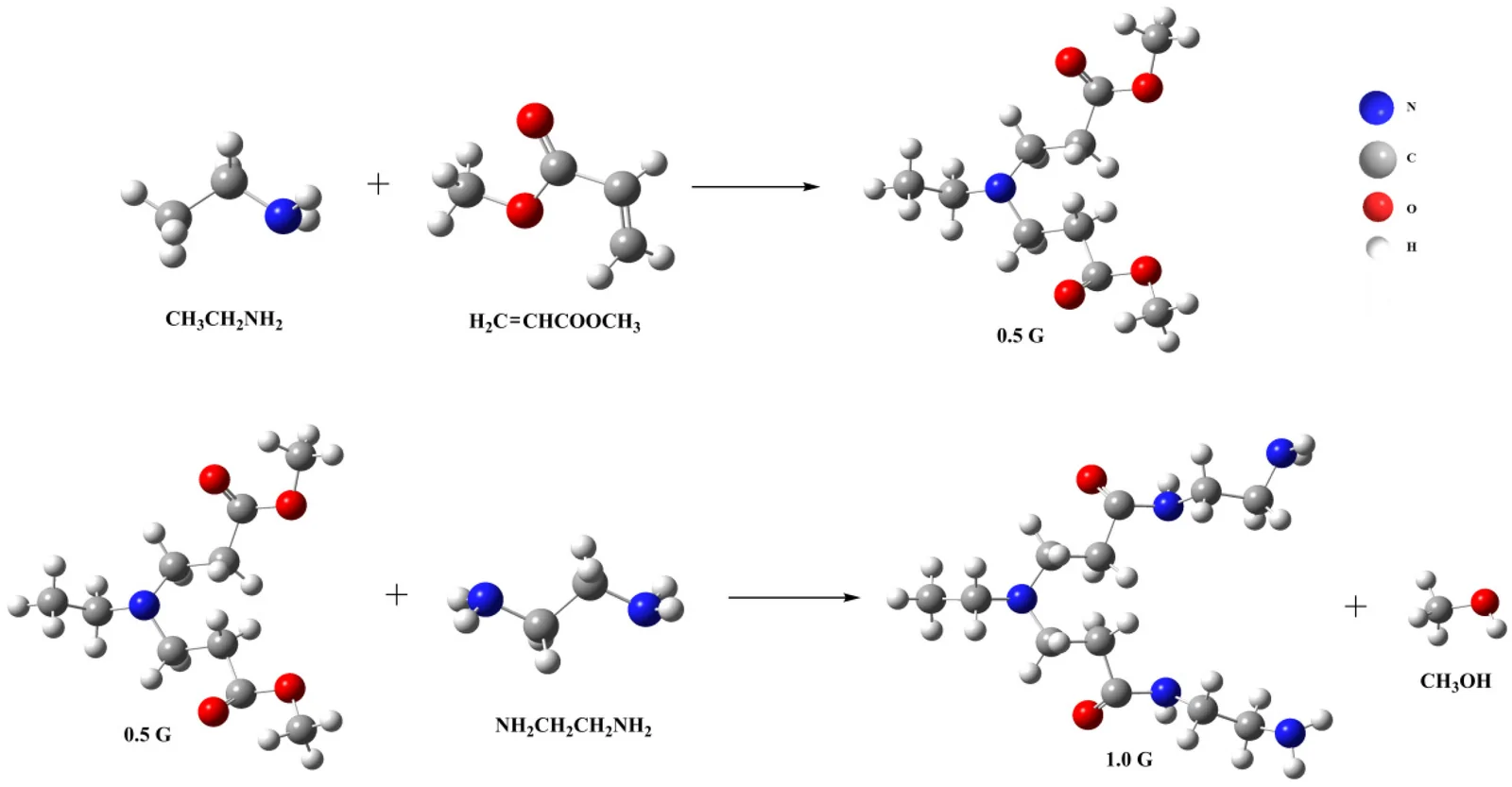
Synthetic routes of 1.0 G series hyperbranched macromolecules.

For the synthesis of C2-1.0 G, ethylamine (4.5 g, 0.1 mol) was added to methanol (10 g), and the mixture was maintained under stirring for 30 min. A methyl acrylate solution (51.6 g, 0.6 mol) was subsequently introduced at 25 °C, after which the reaction mixture was stirred for 24 h. The product was transferred to a rotary evaporator to remove methanol and excess methyl acrylate to give a pale yellow liquid, and a half-generation (0.5 G) hyperbranched broom polymer was obtained. Yield: 21.59 g (99.5%). A solution of 0.5 G hyperbranched broom polymer (10.85 g, 0.05 mol) was added to methanol (40 g), and the mixture was maintained under stirring for 30 min at 25 °C. A EDA solution (51.6 g, 0.6 mol) was subsequently introduced at 25 °C, after which the reaction mixture was stirred for 24 h. Removal of excess EDA under reduced pressure afforded 1.0 G hyperbranched broom polymer as a light-yellow viscous liquid. Yield: 13.48 g (98.8%). The preparation process is illustrated in Figure 12.

### 4.3. Preparation of Hyperbranched Broom Hydrogels

**Figure 13:**
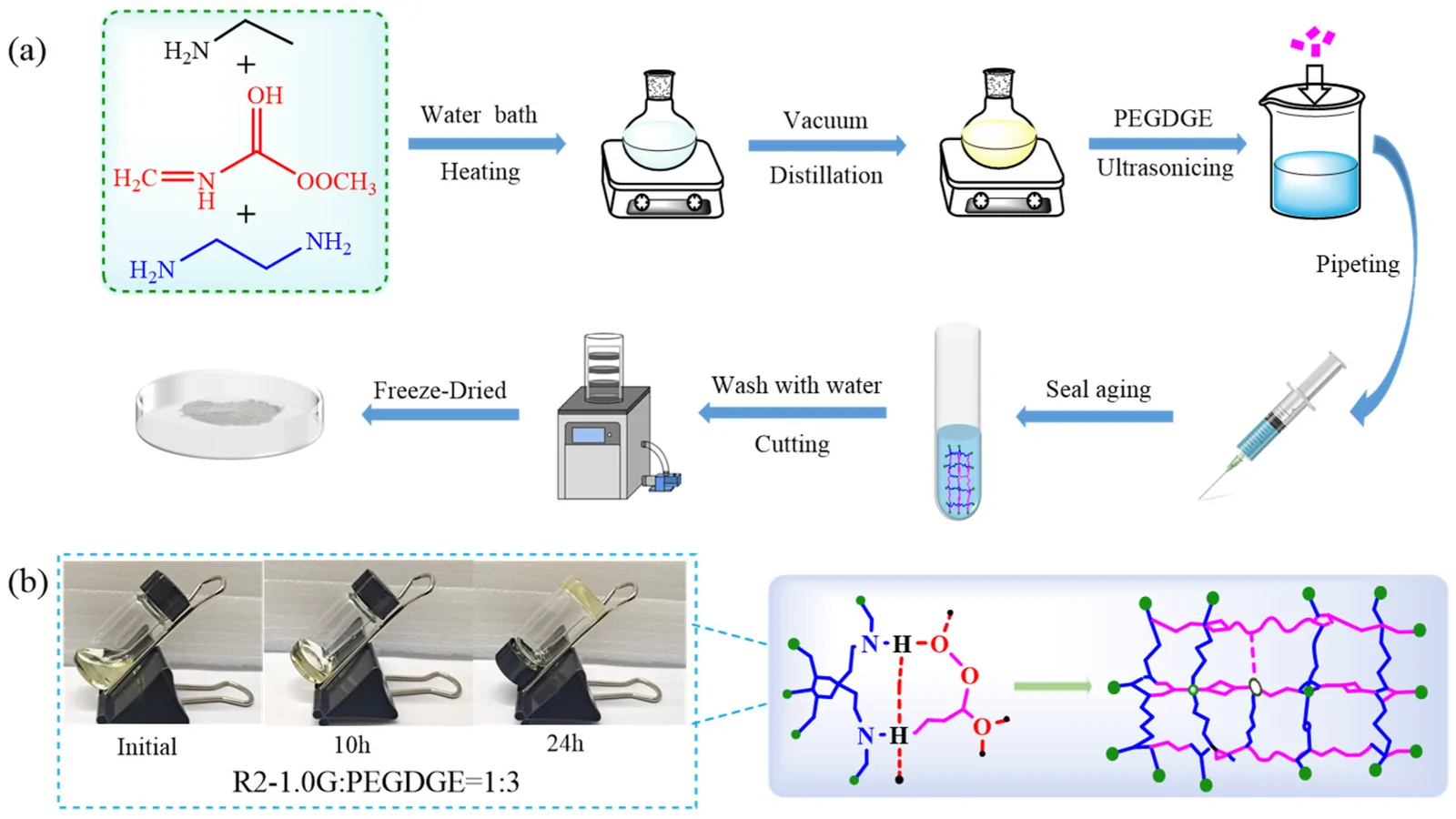
Preparation schematic of hyperbranched hydrogels (**a**) and gelation process (**b**).

C2-1.0 G (0.10 g, 0.37 mmol) and PEGDGE (1.09 g, 1.10 mmol) were dispersed in distilled water (2.79 g), followed by ultrasonication until a uniform aqueous mixture was obtained. The resulting precursor solution was injected into a glass tube with an inner diameter of 10 mm using a syringe. After sealing the tube with a rubber septum, gelation was allowed to proceed in a water bath at 45 °C for 72 h. Upon completion of the reaction, the glass mold was removed from the bath and carefully fractured to recover the cylindrical hydrogel. The hydrogel rods were subsequently sectioned into disks with a thickness of 2 mm. To eliminate residual amines, oligomers, and other unreacted species, the disks were immersed in excess distilled water at room temperature for 7 days, with the washing medium replaced every 24 h. Finally, the fully swollen samples were vacuum-dried at room temperature to constant mass, as illustrated in Figure 13. Similarly, the same applies to gel preparation methods for two other carbon chain lengths.

### 4.4. Characterization

Fourier transform infrared (FTIR) spectra were collected over the wavenumber range of 4000–500 cm^−1^ using an FTIR spectrometer (Bruker, Fällanden, Switzerland) equipped with the KBr pellet technique. Proton nuclear magnetic resonance (^1^H NMR) measurements were conducted at room temperature on a 400 MHz NMR spectrometer (Varian, Palo Alto, CA, USA), using CDCl_3_ as the solvent and tetramethylsilane (TMS) as the internal standard. The contents of carbon (C), hydrogen (H), and nitrogen (N) in the sample were determined using an elemental analyzer (GK-3, Beijing, China). Using water as the solvent, after a homogeneous solution was obtained, it was transferred with a pipette into test tubes, filling each to about two-thirds of its volume. Particle-size distributions were determined with a laser particle-size analyzer (Zetasizer Nano ZS90, Malvern, UK). The surface morphologies of the samples were examined by scanning electron microscopy (SEM, ZEISS Sigma, Oberkochen, Germany) at an accelerating voltage of 10 kV. Prior to imaging, the specimens were coated with a thin gold layer to minimize surface charging. The gel was treated by freeze-drying (Scientz-18N/A, Ningbo, China). This treatment can prevent external drying from breaking the structure of the gel. Thermogravimetric analysis (TGA) was performed using a thermal analyzer (HTG-1, Beijing, China) under a nitrogen atmosphere, with the samples heated from room temperature to 600 °C. Differential scanning calorimetry (DSC) measurements were carried out using a DSC instrument (HSC-1, Beijing, China) under a helium flow at a heating rate of 3 °C /min. The lower critical solution temperature (LCST) was identified from the corresponding endothermic peak.

### 4.5. Rheological Measurements

A hybrid rheometer (DHR-2, TA, New Castle, DE, USA) was used to characterize the viscoelastic behavior of the hydrogel samples. Frequency-sweep measurements were conducted over an angular frequency range of 1–100 rad/s at a constant strain amplitude within the linear viscoelastic region. The storage modulus (G′), loss modulus (G″), and complex viscosity (η*) were recorded. All measurements were performed at room temperature.

### 4.6. Measurement of Swelling Property

For the swelling measurements, hydrogel disks were put into the solution. The swollen gels were taken out at regular time intervals, wiped superficially with filter paper, weighed, and placed again in the same immersion bath. The measurements were continued until a constant weight was reached for each sample. Equation (1) was used to calculate the swelling ratio (*SR_t_*) at a given time, t.

(1) $$SR_{t}/\%=\left(\frac{m_{t}-m_{d}}{m_{d}}\right)\times 100$$

where *m_d_* is the initial weight of the dried gel, and *m_t_* is the weight of the swollen gel at time *t*.

### 4.7. Environmental Response Sensitivity

The environmental response characteristics of hydrogels include temperature response, pH response, and salinity response. In the temperature response test, a xerogel sample was placed in 200 mL of deionized water, and the equilibrium swelling ratio (ESR) of the gel at different temperatures was determined. In the pH response test, glacial acetic acid and aqueous ammonia were used to prepare buffer solutions with set pH values; the sample was soaked in the buffer solution, and the swelling degree of the gel under each condition was calculated based on the mass change before and after vacuum drying at room temperature. The salinity response test systematically evaluated the influence of salinity on hydrogel swelling behavior by placing gel samples in NaCl, CaCl_2_, and MgCl_2_ solutions of different concentrations and recording the mass changes in the gels under different salinity conditions. Equilibrium swelling tests for each condition were performed in triplicate, and the data shown in the figures are the mean values.

### 4.8. Core Plugging Experimental Test

**Figure 14:**
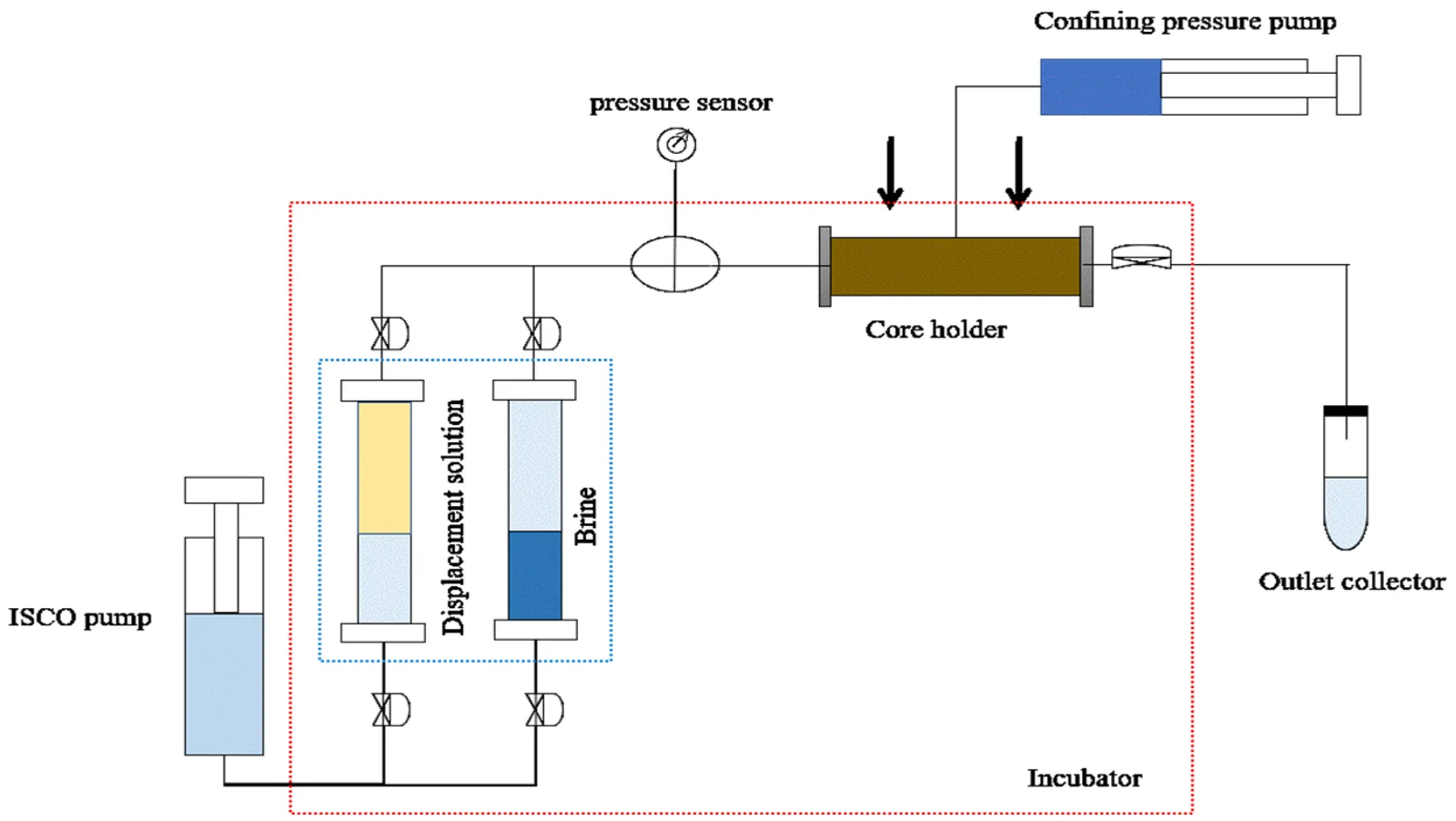
Core displacement device.

The purpose of the core flow experiment is to evaluate the performance and applicability of gel plugging agents. Through indoor core flow experiments, the plugging behavior of gel plugging agents can be intuitively and effectively studied, thus providing an experimental basis for field operations in the oilfield. The experimental setup was constructed following the schematic diagram presented in Figure 14. A 500 mL volume of displacement fluid was prepared at room temperature and magnetically stirred for more than one hour to ensure complete dissolution. After testing the tightness of the experimental setup, the plugging formulation was introduced into the core at an injection volume of 1.0 PV. The core sample was aged at 45 °C for 24 h in a temperature-controlled incubator, and then reversed displacement was carried out with simulated saltwater. The pressure changes were recorded, and the breakthrough pressure was tested. The breakthrough pressure gradient (*P_l_*), permeability (*K_w_*), and plugging ratio were calculated using Equations (2)–(4). The core plugging experiments were conducted in duplicate, and the reported results are the corresponding mean values.

(2) $$P_{l}=\frac{P}{L}$$

(3) $$K_{w}=\frac{Q\mu L}{A(P_{1}-P_{2})}$$

(4) $$E=\frac{K_{1}-K_{2}}{K_{1}}\times 100\%$$

where *P_l_* is the breakthrough pressure gradient, MPa/m; *P* is the breakthrough pressure, MPa; *L* is the core length, cm; *Q* is the volume flow rate, cm^3^/s; *μ* is the fluid viscosity, mPa·s; *A* is the core cross-sectional area, cm^2^; *P*_1_ is the core inlet pressure, MPa; *P*_2_ is the core outlet pressure, MPa; *K*_1_ is the fracture permeability before plugging, mD; *K*_2_ is the fracture permeability after plugging, mD.

## Figures and Tables

**Table 1:** TGA results of the hyperbranched hydrogels with different alkyl chain lengths.

Sample	T_50%_ (°C)	Gel Remaining (%)	T_max_ (°C)
C2HG	406.1	15.1	410.4
C6HG	405.6	11.2	409.4
C8HG	400.4	6.7	408.7

T_50%_: temperature at 50% mass loss. Gel remaining: residual mass of gels. T_max_: maximum mass loss rate temperature.

**Table 2:** Core plugging experimental results.

Sample	Water Measurement Permeability/mD		Breaking the Pressure Gradient/MPa·m^−1^	Plugging Ratio/%
	K_w1_	K_w2_		
C2HG	684.08	0.346	6.065	99.949
C6HG	706.73	0.232	6.541	99.967
C8HG	698.51	0.174	6.953	99.975

## Data Availability

The original contributions presented in this study are included in the article. Further inquiries can be directed to the corresponding author.
